# Pertussis outbreak investigation in Janamora district, Amhara Regional State, Ethiopia: a case-control study

**DOI:** 10.11604/pamj.2019.34.65.19612

**Published:** 2019-10-01

**Authors:** Lezhialem Almaw, Hailemichael Bizuneh

**Affiliations:** 1 Public Health Emergency Management Core Process, Gambella Regional Health Bureau, Gambella, Ethiopia; 2 Epidemiology Unit, Department of Public Health, St. Paul’s Hospital Millennium Medical College, Addis Ababa, Ethiopia

**Keywords:** Bordetella pertussis, outbreak, case control, Ethiopia

## Abstract

**Introduction:**

On April 17/2017 Janamora district, Amhara regional state health officials reported an increasing number of people with a cough. The objectives of this study was to investigate the outbreak, describe risk factors and implement control measures.

**Methods:**

We conducted a community based unmatched 1:1 case-control study April 22-May 10, 2017. We used a probable case definition (≥2 weeks cough with vomiting, apnea, or inspiratory whoop) to identify suspected pertussis cases. Neighbors of cases were considered as controls. We conducted a door-to-door active case search and reviewed medical records, assessed vaccination status by parental interview or vaccination card. We implemented multivariable logistic regression to identify independent factors associated with the outbreak.

**Results:**

We investigated 60 cases and 60 controls. Most (68.3%) of the cases were under the age of 15. The majority (86.6%) of pertussis suspected cases, and 83.4% controls had not received any pertussis vaccine. The overall attack rate was 0.13% and the case fatality rate was 3.3%. The age-specific attack rate for under-five children was 0.33%. Females were more likely to have pertussis (AOR: 2.91; 95% CI: 1.17-7.22), contact with pertussis suspected person (AOR: 6.29; 95% CI: 2.53-15.62) and living in a relatively poorly ventilated house (AOR: 3.01; 95% CI: 1.17-7.70) were also significant risk factors of pertussis.

**Conclusion:**

Weak supplementary immunization activities might have contributed to the outbreak. Treating household contacts and integration of diagnostic laboratory test of pertussis into the local health system is of paramount importance to detect outbreaks early on.

## Introduction

Pertussis, also known as “whooping cough,” is a contagious, acute respiratory illness caused by the gram-negative *Coccobacillus* bacterium *Bordetella pertussis* [1-4] a fastidious, toxin-producing bacillus that causes damage to the respiratory tract and is characterized by intermittent paroxysms (spasms) of severe coughing lasting from 6 to 10 weeks [4, 5]. Pertussis is mainly transmitted by large droplet infection or direct contact with respiratory discharges [4, 6]. Pertussis sometimes lacks fever and classically progresses through three stages [7-10]. Incomplete immunization puts children at greater risk of acquiring the disease [11]. The worldwide increase in vaccination coverage against pertussis has substantially reduced the morbidity and mortality associated with this disease [6, 12, 13]. Over 10 million cases and as many as 400,000 pertussis-related deaths occur annually, with 90% occurring in infants from developing countries [14, 15]. The case-fatality rates in developing countries are estimated to be as high as 4% [16]. In 2015, more than half of all estimated pertussis-related deaths of children <5 years of age occurred in Africa [17]. In Ethiopia, as part the expanded immunization program (EPI) children are vaccinated for pertussis through DTP (diphtheria-pertussis-tetanus) given at 6^th^, 10^th^ and 14^th^ weeks. Even though the vaccination coverage in Ethiopia is increasing, the 2016 Ethiopian Demographic Health Survey (EDHS) showed that in Amhara region, only 46% of children aged 12-23 months received all basic vaccinations including DTP [18]. A study conducted in Ethiopia reported an overall attack rate of 1.3 per 1,000 inhabitants and a case fatality rate of 3.7% [19]. Despite the repeated occurrence of pertussis outbreaks in Ethiopia, data on its risk factors is lacking mainly due to lack of surveillance data, weak surveillance system and absence of diagnostic facilities for confirmation of *Bordetella pertussis* [10]. This study was initiated after a report from the local health office of an increasing number of individuals with cough. The District Health Department was notified on April 17, 2017, and the Zonal Health Office notified the Regional Health Bureau on April 18, 2017. The objective of this investigation was to confirm the existence of an outbreak, identify the potential risk factors and characterize the extent of the outbreak in Janamora District, Amhara region.

## Methods

**Study design and setting:** we conducted an unmatched community based case-control study from April 22 - May 10, 2017. The outbreak occurred in Amhara National Regional State, Janamora district, Bahir Amba kebele (smallest administrative unit) which is one of the 34 rural *kebeles* found in the district. It has a total population of 44,374 of which 22,528 are females, and 6,008 are under five children. The population in the catchment area had one health center with six health professionals and one health post unstaffed without a health extension worker. The vaccination coverage reported from the district was for 74% for Penta (DTP-HepB1-Hib1, OPV1, PCV1, Rota1) 1 (given on 6^th^ week), 69% for Penta 2 (given on 10^th^ week) and 65% for Penta 3 (given on 14^th^ week) for the year 2016/17.

### Case definitions

**Suspected case:** any person with cough, fever and/or coryza and/or headache or any person in whom a clinician suspects pertussis [4].

**Probable case:** person presented with a cough lasting ≥2 weeks, with one or more of the following symptoms: paroxysmal cough or inspiratory whoop or posttussive vomiting and not epidemiologically linked. For infants <1 year only: A case with acute cough illness of any duration with at least one case-defining symptom and apnea (with or without cyanosis) [4].

### Operational definitions

**Vaccinated:** a child who has received at least one dose of pertussis-containing vaccines, and first single dose vaccination status was documented in their log files and/or the mother or the caretaker reported the child received the vaccine.

**Unvaccinated:** a child who has not received any dose of pertussis vaccine and had no record of vaccination on logbooks and the mother or caretaker failed to recall the vaccination status of the child.

**Contact:** spending considerable hours together with coworkers or immediate family members or sleep under the same roof.

**Ventilation of houses:** houses with two or more windows which allow air exchange and assist the spread of bacteria from person to person were classified as having better ventilation. Houses with one or no windows were classified as poor ventilation. The cases were all patients with symptoms of pertussis who came to health facilities and found through a door-to-door active case search by the investigation team. We included cases which presented with the suspected/probable case definition of pertussis that had symptoms from March 06th to 10^th^ May 2017. We found 60 cases from the study area during the outbreak. Of those, 45 were initially documented on a line list and others were interviewed at the health facility and 15 cases were identified by searching for their residence. Controls were selected by a lottery sampling technique as one of the neighbors of the identified cases who had not developed signs and symptoms of pertussis.

**Data collection procedure:** a line list was prepared to gather information about new cases and was maintained until the end of the outbreak. Data for infants were collected by interviewing their mothers or caregivers. Cases and controls were interviewed face to face using a questionnaire that included the factors: socio-demographic data, knowledge about pertussis, clinical features, exposure, treatment and the possible source of infection. We assessed and reviewed surveillance reports in Bahir Amba *kebele* health bureau and obtained vaccination coverage of the district and in the catchment area of the *kebele*. Two epidemiology professionals and two clinical nurses collected the data. The data were registered online list and collected via a structured questionnaire was cross-checked to ascertain and evaluate any inconsistency of the collected data and check completeness.

**Data processing and analysis:** we used SPSS software version 20 to perform bivariate analysis with a p-value of 0.2 as a cut off point for further analysis in a multivariable logistic regression to test the independent association of risk factors with the pertussis at 95% confidence interval and a p-value of 0.05.

**Ethical consideration:** we obtained ethical clearance from St. Paul's Hospital Millennium Medical College, Department of Public Health. Verbal informed consent was obtained from all participants.

## Results

**Demographic characteristics:** a total of 60 pertussis cases were found of which 36 (60%) were female. Majority (68.3%) of the cases were under the age of 15. The median age of the cases and the controls was 6.5 years (range - 9 month to 28 years) and 11.5 years (range - 1 year to 34 years) respectively. The majority (n=56, 93%) of the mothers or caretakers for the cases had no formal education. Most (n=46, 76.6%) of cases and half (n=32, 52.3%) of the controls lived in a house with inadequate ventilation (Table 1).

**Table 1:** Age and sex distribution of the study subjects, Janamora District, Amhara Regional State, Northwest Ethiopia, 2017. (n=120)

Variable	Case (n=60)		Control (n=60)		Total N (%)
Age in year	Male	Female	Male	Female	
<1	2	1	-	-	3 (2.5)
1-4	8	9	6	7	30 (25)
5-9	5	11	7	5	28 (23.3)
10-14	4	7	4	6	21 (17.5)
15-19	5	5	12	7	29 (24.1)
>19	0	3	2	4	9 (7.5)

**Descriptive epidemiology:** the overall attack rate (AR%) was 0.13% and the age-specific attack rate for under-five children was 0.33%. The sex-specific attack rate for females was 0.159% and for males was 0.11%. We identified a total of 60 suspected pertussis cases and 2 deaths were reported. The case fatality rate was 3.3%.

**Date of health facility visit:** of the total 60 pertussis suspected/probable cases, the first case visited the health facility on April 04, 2017 at *Mesha/Cheroleba* area health center, and the peak health facility attendant was seen in April 17 2017, then the number became steady for a couple of weeks. The outbreak spanned a period of 2 months. After the presumed index case was identified, the district health department was notified on April 17, 2017, and the zonal health officials notified the regional health bureau on April 18, 2017.

**Vaccination status:** majority (n=52, 86.6%) of reported pertussis suspected cases and controls (n=50, 83.4%) had not received any pertussis vaccine. Of the 8 cases vaccinated for pertussis, 8 were vaccinated for Penta 1, only 4 were vaccinated for the second dose Penta 2, and none of them were vaccinated for the third dose Penta 3. Among the pertussis cases, most parents or caregivers (n=47, 78.3%) did not know the exact time when vaccination was provided and 19 (31.6%) felt the vaccination would hurt their child. The majority, 55 (91.6%) of the cases reported the health facility was too far, more than half 39 (65%) reported they had to travel more than 2 hours on foot to get health service. The health extension worker deployed to the health post in the *kebele* to provide primary health care, including vaccination to the community was not on her job for more than a year.

**Clinical sign and symptoms:** most (n=49, 83.3%) of the cases, presented with fever and coryza, all (n=60, 100%) had a cough. Of the three infants who were suspected with pertussis, two presented with apnea (Table 2).

**Table 2:** Clinical sign and symptoms, diagnostic criteria for pertussis cases, Jana Amora District, North Gondar, Amhara, Ethiopia, 2017

Symptom	Total (%)
Fever	60 (100)
Coryza	49 (81.6)
Cough	60 (100)
Vomiting after cough	52 (86.6)
Inspiratory whoop	44 (73.3)
Cyanosis after cough	35 (58.3)
Apnea	*
Paroxysmal cough still present	39 (65)

* Out of a total of 3, 2 had apnea

**Knowledge about pertussis:** of all the pertussis cases, 35 (58.3%) said they had not heard of pertussis and close to half (n=28, 46.6%) said pertussis can be transmitted through feco-oral contamination and 16 (26.6%) said it can be transmitted through food, 11 (18%) and 5 (8%) reported contact with pertussis suspected cases and that the disease is airborne respectively. A quarter (n=16, 26.6%) of them said pertussis is vaccine preventable and the rest said they chose local traditional healers to prevent pertussis.

**Analytical epidemiology:** vaccination status was not associated with pertussis. Other factors associated with pertussis on bivariate analysis at a p value 0.2 were further analyzed in multivariate logistic regression to identify independent predictors. Accordingly, females were more likely to develop pertussis (Adjusted Odds Ratio (AOR): 2.91; 95% CI: 1.17-7.22), those who had contact history with pertussis suspected person were more likely to have pertussis (AOR: 6.29; 95%, CI: 2.53-15.62). Similarly, contact with pertussis cases at the workplace, mainly while farming and looking after cattle was also associated with pertussis (AOR: 3.33; 95% CI: 1.28-8.65). People living in houses with poor ventilation were also more likely to develop pertussis (AOR: 3.01; 95% CI: 1.17-7.70) (Table 3).

**Table 3:** Risk factors for pertussis outbreak in Bahir Amba kebele, Jana Amora, Amhara, Ethiopia, 2017

Variables	Options	Case N (%)	Control N (%)	COR+ (95% CI)	AOR (95% CI)
Traveled to other areas	No	23 (38.3)	33 (55)	1	
	Yes	46 (61.7)	27 (45)	1.96 (0.95-4.07)	
Age	≥15	11 (18.4)	20 (33.4)	1	
	<15	49 (81.6)	40 (66.6)	0.44 (0.19-1.04)	
Sex	Male	24 (40)	32 (53.4)	1	1
	Female	36 (60)	28 (46.6)	1.74 (0.83-3.53)	2.91 (1.17-7.22)*
Pertussis prevention method preferred	Vaccination	16(26.7)	24 (40)	1	
	Local traditional healer	44 (73.3)	36 (60)	0.54 (0.25-1.17)	
Ventilation of house	Better ventilated	14 (23.3)	28 (46.7)	1	1
	Poorly ventilated	46 (76.7)	32 (53.3)	2.87 (1.31-6.29)	3.01 (1.17-7.70)*
Contact With a Suspected case	No	14 (23)	36 (60)	1	1
	Yes	46 (77)	24 (40)	4.92 (2.23-10.8)	6.29 (2.53-15.62)*
Contact with a suspected case at work	No	12 (20)	24 (40)	1	1
	Yes	48 (80)	36 (60)	2.66 (1.17-6.03)	3.33 (1.28-8.65)*
Have knowledge of pertussis transmission	Yes	16 (26.7)	24 (40)	1	
	No	44 (73.3)	36 (60)	(0.84-3.96)	

+ Crude Odds Ration

* P value <0.05

**Control measures:** we provided case management and enhanced the surveillance system in kebele. We treated suspected cases with *Azithromycin* and *Erythromycin*. Of the patients who received antibiotics, four were treated for the second time and 35 (58.3%) had not responded totally and had some intermittent cough, and 21 (35%) recovered. We provided mass health education for cases, mothers or caretakers and healthy individuals at the local church, at school, and during our door to door active case search.

## Discussion

The delay in notification of the outbreak in this study area indicates that there was a poor active disease surveillance system at the local and district level. In this study, only 13.4% of cases had received pertussis-containing vaccine which is much lower compared with findings from a similar study (66.7%) [20]. A possible reason might be that the health extension worker who is responsible for notification of outbreaks and providing routine immunization had been off work for almost a year. Moreover, the distance of the health facility from the community might have also hindered the community from seeking care at the nearest facility. Overall pertussis containing vaccination coverage reported from the district was 69.3% which is lower as compared with the coverage in a similar study (90%) [20]. In our study, only 8 (13.3%) and 4 (6.6%) vaccinated for the pertussis-containing vaccine of Penta 1 (given on 6^th^ week) and Penta 2 (given on 10^th^ week) respectively which is considerably lower in contrast with Amhara regions' vaccine coverage [21]. In our study, the overall attack rate of the outbreak was 0.13% (130 cases per 100,000 populations) which is higher compared to findings from similar outbreaks with 5.7 and 1 per 100 000 population respectively [22-24]. Moreover, the case fatality rate among the cases in our study (3.3%) was relatively higher as compared with a similar outbreak (0.17%) [23]. This discrepancy could be attributed to the lack of laboratory technology in the regional state which rendered the challenge to isolate the agent. Moreover, patients visit health facilities very late after falling severely ill which made the early detection and containment of the outbreak slow.

Having a contact history with pertussis suspected person and living in relatively poorly ventilated houses were associated with pertussis, similar to a study conducted in urban Uganda where individuals that were exposed to a coughing individual in the home or neighborhood were more likely to have pertussis [14]. This might be attributed to the fact that the disease is transmitted by droplets or direct contact with discharges from respiratory mucous membranes. This transmission is higher with cases present in the households and even higher in houses with a lower number of windows [25]. In our study, females were nearly three times at a higher risk of contracting pertussis. However, this finding was in contrast to a study which reported odds of getting pertussis disease was not significantly different among sexes [24]. In our study, the possible reason for higher odds of female cases could be that in this particular community, females spend more time working at home and caring for sick children or adults at home, which puts them at a much higher risk of contracting the disease. The limitations in this investigation included that the outbreak was not confirmed by laboratory for a definitive diagnosis. The controls were not matched with respect to possible risk factors and had a ratio of one to one. Furthermore, even though receiving full dose of vaccination is an important risk factor, in this investigation, vaccination cards were not available in some cases and vaccination status was partly determined based on self-report of mothers or care takers which could have been subjected to recall bias.

## Conclusion

The outbreak most likely resulted because of the accumulation of susceptible individuals in the *kebele* due to low routine immunization and weak supplementary immunization activities. This outbreak could have been reduced or even prevented if there was a health extension worker in the area, and timely reporting of cases with a well-functioning surveillance system. Routine and outreach expanded immunization programs need to be strengthened in the area. Health education should be provided extensively to the community by rural health extension workers on ways of pertussis transmission prevention mechanisms including vaccination.

### What is known about this topic

- Extensive evidence exists on the success rate of myringoplasty in relation to functional gain in hearing post-surgery worldwide;
- International evidence exists on the efficacy of myringoplasty surgery in terms of audiological improvement worldwide;
- Vaccination coverage against pertussis is increasing.

### What this study adds

- Pertussis outbreak is affected by absence of local health extension workers who provide routine immunization;
- Poorly ventilated housing exacerbated pertussis transmission;
- Women were prone to contracting pertussis as they provide care for pertussis cases at home.

## Competing interests

The authors declare no competing interests.
